# Contour similarity and its implication on inverse prostate SBRT treatment planning

**DOI:** 10.1002/acm2.13809

**Published:** 2022-10-27

**Authors:** Chenyu Yan, Bingqi Guo, Rahul Tendulkar, Ping Xia

**Affiliations:** ^1^ Department of Radiation Oncology Cleveland Clinic Foundation Cleveland Ohio USA

**Keywords:** auto‐segmentation, contour similarity, prostate SBRT

## Abstract

**Purpose:**

Success of auto‐segmentation is measured by the similarity between auto and manual contours that is often quantified by Dice coefficient (DC). The dosimetric impact of contour variability on inverse planning has been rarely reported. The main aim of this study is to investigate whether automatically generated organs‐at‐risk (OARs) could be used in inverse prostate stereotactic body radiation therapy (SBRT) planning and whether the dosimetric parameters are still clinically acceptable after radiation oncologists modify the OARs.

**Methods and materials:**

Planning computed tomography images from 10 patients treated with SBRT for prostate cancer were selected and automatically segmented by commercially available atlas‐based software. The automatically generated OAR contours were compared with the manually drawn contours. Two volumetric modulated arc therapy (VMAT) plans, autoRec‐VMAT (where only automatically generated rectums were used in optimization) and autoAll‐VMAT (where automatically generated OARs were used in inverse optimization) were generated. Dosimetric parameters based on the manually drawn PTV and OARs were compared with the clinically approved plans.

**Results:**

The DCs for the rectum contours varied from 0.55 to 0.74 with a mean value of 0.665. Differences of *D*_95_ of the PTV between autoRec‐VMAT and manu‐VMAT plans varied from 0.03% to −2.85% with a mean value of −0.64%. Differences of *D*_0.03cc_ of manual rectum between the two plans varied from −0.86% to 9.94% with a mean value of 2.71%. *D*_95_ of PTV between autoAll‐VMAT and manu‐VMAT plans varied from 0.28% to −2.9% with a mean value −0.83%. Differences of *D*_0.03cc_ of manual rectum between the two plans varied from −0.76% to 6.72% with a mean value of 2.62%.

**Conclusion:**

Our study implies that it is possible to use unedited automatically generated OARs to perform initial inverse prostate SBRT planning. After radiation oncologists modify/approve the OARs, the plan qualities based on the manually drawn OARs are still clinically acceptable, and a re‐optimization may not be needed.

## 1 INTRODUCTION

Segmentation of medical images aims to locate anatomic structures and delineate their boundaries on a digital source such as computed tomography (CT) or magnetic resonance imaging scans, which is a crucial step in radiation treatment planning. In most cases, segmentation is done by an experienced clinical expert, and this process is often time‐consuming^1^ and subject to interobserver variations.^2^, ^3^ To improve efficiency and reduce the workload of clinicians, auto‐segmentation has been introduced to radiation therapy and has been an active area of research for many years.^4^, ^5^, ^6^, ^7^, ^8^ Traditional auto‐segmentation algorithms^4^, ^5^, ^6^, ^7^, ^8^ used in radiation therapy are mostly based on the direct analysis of image content and properties such as voxel intensities and/or image gradient, and their performance is limited by insufficient soft tissue contrast of CT data that makes it harder to accurately delineate critical organ boundaries. Such limitation motivated the search for new segmentation algorithms powered by prior‐knowledge, and one of such algorithms is the atlas‐based segmentation. Atlas‐based segmentation applies prior knowledge by using a reference image, also referred to as atlas (image), in which structures of interest are already segmented.^9^ The segmentation of corresponding structures in a new target image is obtained by finding the optimal transformation between the atlas image and the target image. Different techniques of transformation such as demons registration,^10^ block‐matching,^11^ and B‐spline^12^ registration have been used in radiotherapy.^13^, ^14^, ^15^ The choice of a suitable and robust error metric,^16^ such as mutual information, is very important in handling image noise or changes of image contrast.

To speed up the contouring process and improve consistency among observers, a number of atlas‐based contouring products have become commercially available recently. Most of the products use a form of atlas‐based contouring and are facilitated by a model‐based method, but these are generally limited to certain organs‐at‐risk (OARs).^17^ Performance of different automatic contouring tools applied to various sites has been reported for head and neck, breast, abdomen, and lung.^17^, ^18^, ^19^, ^20^, ^21^ Most recently, convolutional neural networks^22^—a concept from the field of deep learning—have been applied to auto‐segment head/neck CT images. Dice coefficient (DC) has also been used to train a deep neural network to automatically segment CT images from breast cancer patients^23^ for radiation therapy, and the author concluded that their algorithm has a significant impact on the workload of clinical staff and on the standardization of care. The performance of the auto‐segmentation or deformable image registration algorithms were often evaluated by comparing manual contours with automatically generated contours,^13^, ^22^, ^24^, ^25^, ^26^, ^27^, ^28^ and the similarities between the two set of contours were quantified by mathematical quantities. Among all of the metrics used to measure the similarity between two contours, DC defined as $2\times V_{1}\cap V_{2}/\left(V_{1}+V_{2}\right)$, which describes the intersection of contour volume *V*_1_ and *V*_2_ divided by the average volume, is one of the most popular criteria. DC has a value of 1, when two contours completely overlap with each other, and has a value of 0, when two contours are entirely disjoint (no overlap). Another popular mathematical quantity is used to measure the contour differences, Hausdorff distance (HD) ( $d_{H}$), which is defined as

$$d_{H}\left(A,B\right)=\text{ma}x_{a\in A}\text{mi}n_{b\in B}\left\|a-b\right\|$$

where *A* and *B* are the two contours, which were also calculated for selected contours.

Even though the similarity between two contours could be quantitatively measured by DC or HD, the implication of the similarity on inverse treatment planning has been rarely reported. In modern radiation therapy, intensity‐modulated radiation therapy (IMRT) and volumetric modulated arc therapy (VMAT) are gaining popularity, especially in stereotactic body radiation therapy (SBRT). These techniques use inverse treatment planning to deliver high conformal doses to tumors while minimizing doses to critical surrounding organs. Inverse planning is substantially different from the traditional forward planning even though the two types share some common procedures. Compared with forward planning, inverse planning is nonintuitive and the iterative optimization process is like a black‐box to the user. Consequently, the impact of the contour variations of critical organs, which is often quantified by DC, on the plan quality is not well understood. Therefore, the main aim of this study is to evaluate the impact of contour variability on inverse prostate SBRT planning and investigate whether unedited OARs could be used to generate plans with quality similar to the one optimized with manual OARs. In most clinics, IMRT/VMAT planning will not start until radiation oncologists approve the OARs. In this study, we want to explore whether the OARs automatically generated by commercially‐available software could be used to optimize IMRT/VMAT plans before physicians’ final approval is given. Our study is designed to evaluate whether after radiation oncologists edit/approve OARs, the plan qualities measured by the manually drawn OARs are still clinically acceptable.

## 2 MATERIALS AND METHODS

Planning CT images of 20 prostate patients, who underwent spaceOAR and fiducial marker placement for SBRT, were manually segmented by our experienced experts and used to build template for atlas‐based segmentation algorithm that recently became available in our clinics. On these images, OARs, such as the rectum, bladder, seminal vesicle, and penile bulb as well as prostate and PTV, were manually contoured. CT images from another 10 prostate patients, who underwent spaceOAR and fiducial marker placement for SBRT, were used as target images, and all the critical organs were automatically generated by applying the atlas‐based auto‐segmentation algorithm.

For each of the 10 prostate patients, OARs and PTV were manually drawn by an experienced radiation oncologist, and manu‐VMAT plans, based on the manual contours, were optimized and reviewed by at least two physicians before its clinical use. All 10 plans were VMAT plans with 2 full arcs except for one large patient with 4 partial arcs to avoid collision. PTV, rectum, bladder, spaceOAR, seminal vesicle, and penile bulb were drawn by a physician before optimization starts. During the optimization process, 10 plans used rectum and bladder to create OAR dose constraints and 4000 cGy was prescribed to PTV except for 1 patient for which 3625 cGy was prescribed. In addition to rectum and bladder, 4 of the 10 plans used ring structures (which include all tissues 1 or 2 cm away from the PTV) in objective functions to control dose spillage to other normal tissues such as small bowl and femur head.

To evaluate the impact of contour variability on inverse planning, we first replaced manually drawn rectum with automatically generated rectum and re‐optimized the plans. The plans optimized with automatically generated rectum are denoted as autoRec‐VMAT. Another plan, denoted as autoAll‐VMAT, was generated by replacing all the manually drawn OARs (rectum, bladder) except for the ring structures with automatically generated OARs, and the plans were re‐optimized again. Both autoRec‐VMAT and autoAll‐VMAT plans were optimized from scratch that means the beams were reset before optimization starts. In both cases, beam arrangements are the same as original plans, and the dosimetric constraints and the weight of the objective were adjusted to achieve the best possible plans. As the re‐optimization process started from scratch, the plan parameters’ differences reflect the impact of OARs contours on the inverse planning process. To quantify the contour variability, DCs between the manual and auto‐OARs were evaluated, and HDs between rectums were also calculated to better understand the impact of contour variability.

**FIGURE 1:**
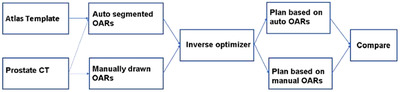
The workflow of our study scheme to evaluate the impact of Dice coefficient (DC) on inverse plan quality

The dosimetric parameters based on the manually drawn contours were compared among manu‐VMAT, autoRec‐VMAT, and autoAll‐VMAT plans. Dosimetric parameters, such as *D*_95_ of PTV and dosimetric parameters for manually drawn rectum and bladder, were compared among the three plans. *D*_0.03cc_ and *D*_20_ of bladder were mainly used to evaluate the plan quality by our radiation oncologist. In our clinics, *D*_95_ of PTV is generally required to be no less than 3625 cGy to achieve proper tumor control. To control the risk of complications, *D*_0.03cc_ of bladder is no more than 4000 cGy, and *D*_20_ of bladder is expected to be less than 1830 cGy (and the secondary goal is *D*_20_ < 3000 cGy). For rectum, *D*_0.03cc_ of anterior rectum, *D*_3cc_ of lateral rectum, and *D*_0.03cc_ of posterior rectum were used by our physicians. Generally, *D*_0.03cc_ of anterior rectum is expected to be less than 3900 cGy, *D*_3cc_ of lateral rectum is less than 2000 cGy, and *D*_0.03cc_ of posterior is expected to be less than 1900 cGy. These dosimetric goals are desired and could be adjusted by our radiation oncologists during plan review to achieve a balance between PTV coverage and OAR dose limits. It is worth emphasizing that even though automatically generated contours were used in inverse optimization process, only manually drawn contours were used to evaluate the plan qualities. Figure 1 shows the workflow of our study scheme.

## 3 RESULTS

### 3.1 DC and PTV *D*_95_

The DC for rectum varies from 0.55 to 0.74 with a mean value of 0.665, whereas the HD varies from 1.4 to 5 cm with a mean value of 2.75 cm. Differences of PTV *D*_95_ varies from 0.98% to −2.85% with a mean value of −0.64% between manu‐VMAT and autoRec‐VMAT plans. The percentage difference was calculated via 1 − (*D*_95_)_auto_/(*D*_95_)_manual_, where (*D*_95_)_auto_ and (*D*_95_)_manual_ are the PTV *D*_95_s from autoRec‐VMAT and manu‐VMAT plans, respectively. Negative percentage difference means (*D*_95_)_auto_ > (*D*_95_)_manual_, whereas positive difference implies (*D*_95_)_manual_ > (*D*_95_)_auto_. For 7 out of the 10 cases, *D*_95_ of autoRec‐VMAT plan is greater than the *D*_95_ of manu‐VMAT plan with largest difference being −2.85% (Patient 7). For the other three cases, *D*_95_ of autoRec‐VMAT plan is less than the *D*_95_ of manu‐VMAT plan with largest difference being 0.98% (Patient 4). Table 1 summarizes DCs, the *D*_95_s and their differences for the two plans. Generally, the absolute differences between the two *D*_95_s are less than 3% and *D*_95_ values of the plan optimized with automatically generated rectum are higher than the *D*_95_ values of the plan optimized with manually drawn rectum. Table 2 shows the PTV *D*_95_ differences between autoAll‐VMAT and manu‐VMAT plans. DCs of bladders are also shown in Table 2, and they vary from 0.73 to 0.91 with an average of 0.83 and standard deviation of 0.07. PTV *D*_95_ differences vary from 0.79% to −2.9% with a mean value of −0.84%. PTV *D*_95_ for autoAll‐VMAT (plan optimized with automatically generated OARs) plans are close or slightly better than the plan optimized with manual OARs.

**TABLE 1:** Dice coefficient (DC) and the *D*_95_ for the two plans, autoRec‐volumetric modulated arc therapy (VMAT) and manu‐VMAT

	DC rectum	HD rectum (cm)	*D*_95_ of autoRec‐VMAT (cGy)	*D*_95_ of manu‐VMAT (cGy)	Percentage difference
Patient 1	0.74	1.4	3842	3843	0.03
Patient 2	0.67	2.5	4062	4052	−0.42
Patient 3	0.74	2.6	4028	4003	−0.62
Patient 4	0.69	2.3	3956	3995	0.98
Patient 5	0.72	2.3	4227	4216	−0.26
Patient 6	0.58	1.9	4072	4016	−1.39
Patient 7	0.58	3.7	4117	4003	−2.85
Patient 8	0.70	5	4091	4054	−0.91
Patient 9	0.55	2.8	4089	4029	−1.49
Patient 10	0.69	3	4020	4041	0.52

*Note*: The differences between the two *D*_95_s are also shown.

Abbreviation: HD, Hausdorff distance.

**TABLE 2:** PTV *D*_95_ for the two plans, autoAll‐volumetric modulated arc therapy (VMAT) and manu‐VMAT

	DC bladder	*D*_95_ of autoAll‐VMAT (cGy)	*D*_95_ of manu‐VMAT (cGy)	Percentage difference
Patient 1	0.90	3897	3843	−1.41
Patient 2	0.89	4065	4052	−0.49
Patient 3	0.86	4037	4003	−0.85
Patient 4	0.91	3984	3995	0.28
Patient 5	0.88	4226	4216	−0.24
Patient 6	0.78	4072	4016	−1.39
Patient 7	0.83	4119	4003	−2.9
Patient 8	0.81	4064	4054	−0.25
Patient 9	0.72	4105	4029	−1.89
Patient 10	0.73	4009	4041	0.79

Abbreviation: DC, Dice coefficient.

**FIGURE 2:**
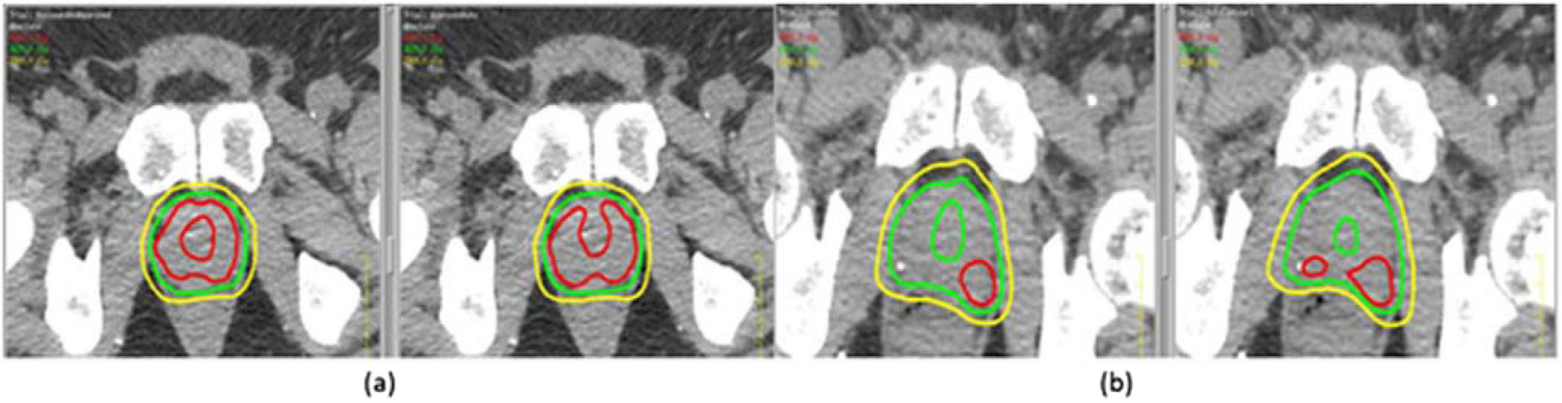
Dose distributions for autoRec‐volumetric modulated arc therapy (VMAT) (left of (a) and (b)) and manu‐VMAT (right of (a) and (b)) plans. The prescribed doses are 3625 cGy (a) and 4000 cGy (b), whereas Dice coefficients (DCs) are 0.74 (a) and 0.55 (b), respectively

Figure 2 compares the dose distributions between autoRec‐VMAT and manu‐VMAT plans. The prescribed doses are 3625 cGy (a) and 4000 cGy (b), whereas DCs for the two cases are 0.74 (a) and 0.55 (b). The isodose distributions based on auto segmented rectum (on the left of (a) and (b)) and manual rectum (on the right of (a) and (b)) are shown side‐by‐side for comparison. By visual inspection, the hot spots (red), coverage of prescribed isodose line (green), and the 80% of prescribed isodose line (yellow) look very similar.

### 3.2 Dosimetric parameters of manual rectums and bladders

Table 3 summarizes the *D*_0.03cc_ of anterior rectum for autoRec‐VMAT and manu‐VMAT plans. The percentage differences between the two *D*_0.03cc_s are also listed. The percentage differences between the two *D*_0.03cc_s range from −0.9% to 9.9% with an average of 2.7%. Similarly, the percentage difference is calculated via 1 − (*D*_0.03cc_)_auto_/(*D*_0.03cc_)_manual_. In most cases, *D*_0.03cc_ for the autoRec‐VMAT plan is less than the *D*_0.03cc_ for the manu‐VMAT plan except for Patients 4 and 10. Generally, manual rectum *D*_0.03cc_ for the autoRec‐VMAT plan is close or less than the *D*_0.03cc_ of manu‐VMAT plan, even though the DC varies from 0.55 to 0.74.

**TABLE 3:** *D*_0.03cc_ of manually drawn anterior rectum for the autoRec‐volumetric modulated arc therapy (VMAT) and manu‐VMAT plans

	Manual rectum *D*_0.03cc_ for autoRec‐VMAT plan (cGy)	Manual rectum *D*_0.03cc_ for manu‐VMAT plan (cGy)	Percentage difference
Patient 1	3270	3399	3.8
Patient 2	3345	3473	3.7
Patient 3	3614	3666	1.4
Patient 4	4135	4130	−0.1
Patient 5	2709	2814	3.7
Patient 6	3495	3517	0.6
Patient 7	3739	3812	1.9
Patient 8	2590	2670	3.0
Patient 9	3235	3592	9.9
Patient 10	4237	4201	−0.9

*Note*: The differences between the two *D*_0.03cc_s are also shown.

Other than *D*_0.03cc_ of the anterior rectum, *D*_3cc_ of lateral rectum and *D*_0.03cc_ of posterior rectum are often used to evaluate the plan quality in our clinics. Therefore, *D*_3cc_ and *D*_0.03cc_ of lateral and posterior rectum achieved by the two plans (autoRec‐VMAT and manu‐VMAT) are listed in Tables 4 and 5. For all patients, *D*_3cc_s of autoRec‐VMAT plans are less than the *D*_3cc_ of manu‐VMAT plans. *D*_0.03cc_s of posterior rectums for autoRec‐VMAT plans are mostly less than the *D*_0.03cc_ of posterior rectums for manu‐VMAT plans except for Patients 1 and 7.

**TABLE 4:** *D*_3cc_ of manually drawn lateral rectum for autoRec‐volumetric modulated arc therapy (VMAT) and manu‐VMAT plans and their percentage differences

	Manual lateral rectum *D*_3cc_ for autoRec‐VMAT plan (cGy)	Manual lateral rectum *D*_3cc_ for manu‐VMAT (cGy)	Percentage difference
Patient 1	813	1030	21.07
Patient 2	636	988	35.63
Patient 3	1190	1646	27.7
Patient 4	1235	1354	8.79
Patient 5	393	442	11.09
Patient 6	945	1083	12.74
Patient 7	1549	1583	2.15
Patient 8	541	705	23.26
Patient 9	962	1121	14.18
Patient 10	1688	1837	8.11

**TABLE 5:** *D*_0.03cc_ of manually drawn posterior rectum for autoRec‐volumetric modulated arc therapy (VMAT) and manu‐VMAT plans and their percentage differences

	Manual posterior rectum *D*_0.03cc_ for autoRec‐VMAT plan (cGy)	Manual posterior rectum *D*_0.03cc_ for manu‐VMAT (cGy)	Percentage difference
Patient 1	1282	1269	−1.02
Patient 2	749	1076	30.39
Patient 3	1341	1357	1.18
Patient 4	1015	1247	18.6
Patient 5	366	434	15.67
Patient 6	1131	1275	11.29
Patient 7	1391	1381	−0.72
Patient 8	635	689	7.84
Patient 9	1117	1257	11.14
Patient 10	1423	1820	21.81

Table 6 lists the *D*_0.03cc_ of anterior rectum for autoAll‐VMAT and manu‐VMAT plans. Tables 7 and 8 summarize *D*_3cc_ of lateral rectum and *D*_0.03cc_ of posterior rectum for the two plans. Our results show that *D*_0.03cc_ and *D*_3cc_ to manually drawn rectums from autoAll‐VMAT plans were either close or better than those from manu‐VMAT plans. Tables 9 and 10 compare *D*_0.03cc_ and *D*_20_ of bladder between autoAll‐VMAT and manu‐VMAT plans.

**TABLE 6:** *D*_0.03cc_ of manually drawn anterior rectum for autoAll‐volumetric modulated arc therapy (VMAT) and manu‐VMAT plans and their percentage differences

	Manual anterior rectum *D*_0.03cc_ for autoAll‐VMAT plan (cGy)	Manual anterior rectum *D*_0.03cc_ for manu‐VMAT plan (cGy)	Percentage difference
Patient 1	3268	3399	3.85
Patient 2	3342	3473	3.77
Patient 3	3610	3666	1.53
Patient 4	4126	4130	0.10
Patient 5	2687	2814	4.51
Patient 6	3487	3517	0.85
Patient 7	3556	3812	6.72
Patient 8	2587	2670	3.11
Patient 9	3500	3592	2.56
Patient 10	4233	4201	−0.76

**TABLE 7:** *D*_3cc_ of manually drawn lateral rectum autoAll‐volumetric modulated arc therapy (VMAT) and manu‐VMAT plans and their percentage difference

	Manual lateral rectum *D*_3cc_ for autoAll‐VMAT plan (cGy)	Manual lateral rectum *D*_3cc_ for manu‐VMAT plan (cGy)	Percentage difference
Patient 1	828	1030	19.61
Patient 2	640	988	35.22
Patient 3	1224	1646	25.64
Patient 4	1193	1354	11.89
Patient 5	392	442	11.31
Patient 6	938	1083	13.39
Patient 7	1546	1583	2.34
Patient 8	542	705	23.12
Patient 9	362	1121	67.71
Patient 10	1722	1837	6.26

**TABLE 8:** *D*_0.03cc_ of manually drawn posterior rectum for autoAll‐volumetric modulated arc therapy (VMAT) and manu‐VMAT and their percentage differences

	Manual posterior rectum *D*_0.03cc_ for autoAll‐VMAT plan (cGy)	Manual posterior rectum *D*_0.03cc_ for manu‐VMAT plan (cGy)	Percentage difference
Patient 1	1287	1269	−1.42
Patient 2	748	1076	30.48
Patient 3	1337	1357	1.47
Patient 4	857	1247	31.28
Patient 5	366	434	15.67
Patient 6	1126	1275	11.69
Patient 7	1390	1381	−0.65
Patient 8	626	689	9.14
Patient 9	1073	1257	14.64
Patient 10	1439	1820	20.93

**TABLE 9:** Manually drawn bladder *D*_0.03cc_ percentage differences between autoAll‐volumetric modulated arc therapy (VMAT) and manu‐VMAT plans

	Manual bladder *D*_0.03cc_ for autoAll‐VMAT plan (cGy)	Manual bladder *D*_0.03cc_ for manu‐VMAT plan (cGy)	Percentage difference
Patient 1	3806	3824	0.47
Patient 2	3861	3873	0.31
Patient 3	3856	3891	0.90
Patient 4	4036	4162	3.03
Patient 5	3880	3949	1.75
Patient 6	3999	3954	−1.14
Patient 7	3767	3784	0.45
Patient 8	3786	3895	2.80
Patient 9	3920	3892	−0.72
Patient 10	4031	4174	3.43

**TABLE 10:** Manually drawn bladder *D*_20_ percentage differences between autoAll‐volumetric modulated arc therapy (VMAT) and manu‐VMAT plans

	Manual bladder *D*_20_ for autoAll‐VMAT plan (cGy)	Manual bladder *D*_20_ for manu‐VMAT plan (cGy)	Percentage difference
Patient 1	890	896	0.67
Patient 2	1203	1192	−0.92
Patient 3	1648	1984	16.94
Patient 4	985	1099	10.37
Patient 5	1449	1431	−1.26
Patient 6	1280	1225	−4.49
Patient 7	2249	2485	9.5
Patient 8	716	801	10.61
Patient 9	228	307	25.73
Patient 10	1934	1909	−1.31

**FIGURE 3:**
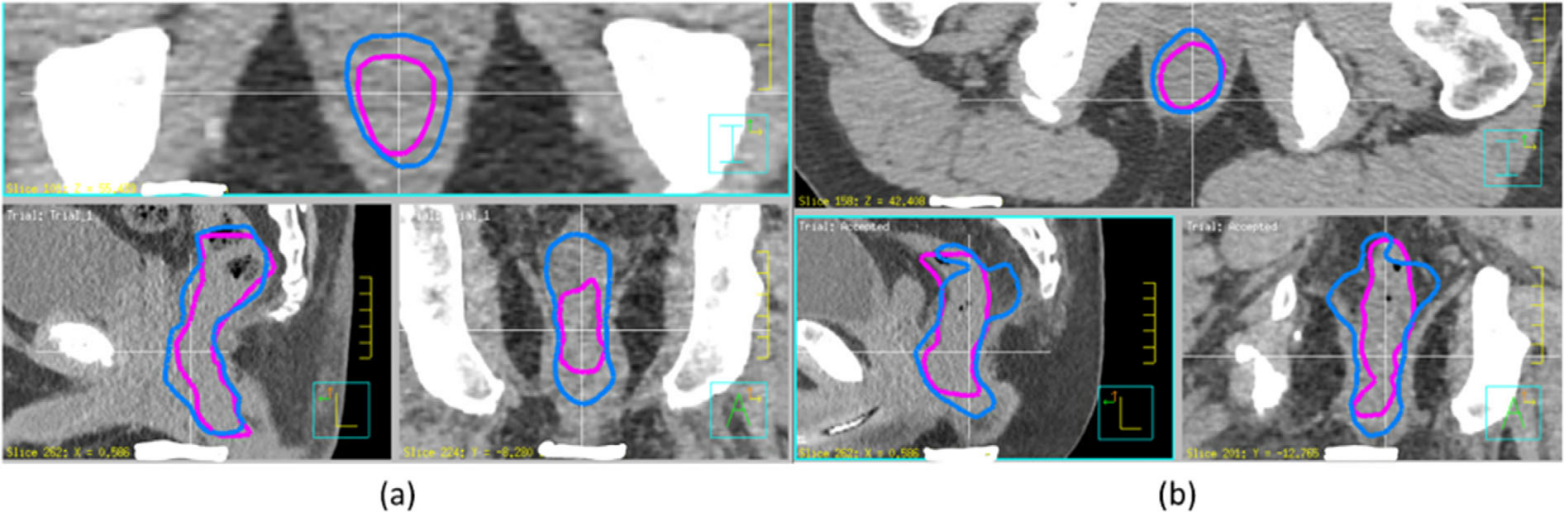
Automatically generated rectum (blue) and manually drawn rectum (purple) are shown in three views. Dice coefficient (DC) for picture (a) is 0.74 (Patient 1), whereas the DC for the picture (b) is 0.55 (Patient 9)

**FIGURE 4:**
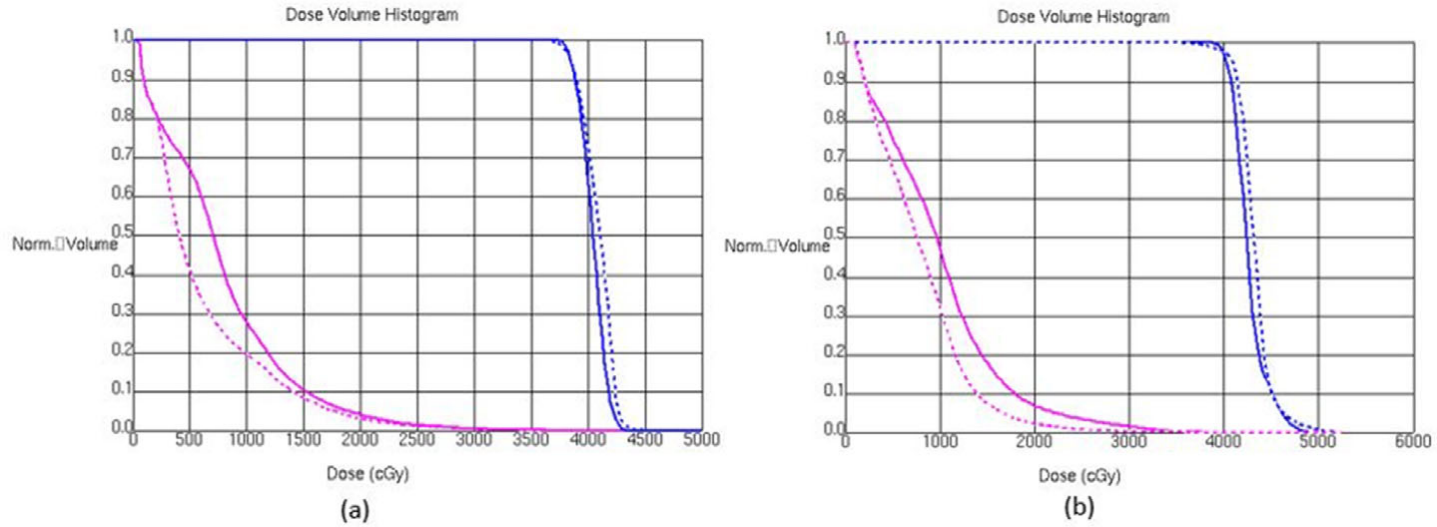
DVHs for autoRec‐volumetric modulated arc therapy (VMAT) (dashed) and manu‐VMAT (solid) plans. PTV (blue) and manually drawn rectum (purple) DVHs are shown. Dice coefficient (DC) for picture (a) is 0.74 (Patient 1), whereas the DC for the picture (b) is 0.55 (Patient 9).

Figure 3 compares automatically segmented rectum (blue) and manually drawn rectum (purple) in three views for the two patients with minimum (Patient 9 (a)) and maximum (Patient 1 (b)) DC. The two rectums shown in Figure 3a are visually more conformal with each other; however, the two rectums in Figure 3b show larger disparities in posterior and lateral regions. Figure 4 compares the DVHs of PTV (blue) and manually drawn rectums (purple) for the plans optimized with automatically segmented rectum (dashed lines) and manually drawn rectum (solid lines), respectively. Similar to Figure 3, Figure 4a compares DVHs for the patient with maximum DC (Patient 1) and Figure 4b shows the DVHs for the patient with minimum DC (Patient 9). Generally, the DVHs are very close to each other and rectum doses are lower for the autoRec‐VMAT plan than the manu‐VMAT plan.

## 4 DISCUSSION

The promise of tremendously reducing the cost and improving efficiency in radiation therapy motivated the search and evaluation of automatic treatment planning algorithms^29^, ^30^; however, auto‐segmentation has been a bottleneck in automatic treatment planning. Consequently, the development of fully automatic segmentation algorithms for radiation therapy has been an interest area of research for many years.^6^, ^14^, ^15^, ^22^, ^27^ Due to low contrast of soft tissue and image artifacts, the performance of the algorithms is still suboptimal, which prevented the widespread use of the auto segmentation tools in clinics. Most of the researchers use contour similarity, which is quantitatively measured by DC between automatically segmented contours and manual contours, to evaluate the performance of the algorithms. However, contour similarity may not fully reveal the impact of contour variability on radiation therapy that, very likely, depends on the specific applications. The implication of contour similarity to a specific application in radiation therapy should be investigated case by case. Therefore, this study intends to evaluate the impact of contour similarity on the applicability of unedited OAR in inverse prostate SBRT radiation planning.

SBRT is a radiation treatment modality that combines a high degree of targeting accuracy and reproducibility with very high doses of extremely precise in short courses. Some clinical studies^31^, ^32^ have indicated that prostate SBRT has shown no difference in late toxicity, patient reported quality of life, or tumor control when compared with conventional external beam radiation therapy (78 Gy in 39 fractions). These studies suggest that prostate SBRT is a safe and effective treatment modality for low‐to‐intermediate risk prostate cancer. Inverse treatment planning techniques, such as IMRT and VMAT, have been a dominant tool in generating highly conformal plans for SBRTs that maximize doses to the target while sparing critical organs. Due to its nonintuitive nature of inverse planning process, the correlation between contour and the planning process is not straightforward and needs careful study to better understand the impact of contour similarity on plan quality. Our study shows that even though DC, which is often used to quantitatively measure contour similarity, can vary from 0.55 to 0.74; these contour variabilities do not prevent us from using the automatically generated OARs to generate clinically accepted plans as shown in Tables 1, –10. This information could be very helpful for us to understand how to use the auto‐segmentation tools effectively in radiation therapy. Lustberg et al.^33^ evaluated atlas‐based and deep learning contouring for lung cancer and concluded that total median time saved was 7.8 min for atlas‐based contouring and 10 min for deep learning contouring. They conclude that user adjustment of software generated contours is a viable strategy to reduce contouring time of OARs for lung radiotherapy. Our results imply that the amount of work of adjusting automatically generated contours could possibly be further reduced. In our clinics, planning will not start until radiation oncologists approve all the OARs and our results imply that planning could start with the automatically generated OARs and the final plan may not need to be re‐optimized after radiation oncologists modify the OARs. This could help us optimize planning workflow and further improve efficiency in clinics.

Intra‐observer and interobserver variabilities (IOV) in contouring have been well studied and reported^34^, ^35^ in literature, and a framework to use future studies evaluating IOV is recommended.^35^ Most auto‐segmentation algorithms measure the performance of the algorithm by comparing auto‐generated contours with expert segmentations^25^, ^27^ and the impact of the inherent contour variability on its application in radiation therapy should be well understood before its use in clinics. Our results show that when used for inverse prostate SBRT treatment planning, the dosimetric impact of OARs contour variability on the plan quality is limited, and this information could be very helpful in developing automatic adaptive radiation therapy scheme.

## 5 CONCLUSION

This study implies that contour similarity alone might not be a good indicator of the usefulness of the automatically generated contours in radiation therapy; therefore, it should not be the only metrics to evaluate the performance of the auto‐segmentation algorithms. In addition, contour variations might be inherent and could be unavoidable; hence, better understanding of the impact of contour variation on radiation therapy could provide some guidance for us to further improve the auto‐segmentation algorithms. Our study implies that unedited OAR contours could be used for inverse prostate SBRT planning to generate clinically acceptable plans when evaluated according to the manual contours. In our clinical practice, OARs need to be approved by radiation oncologist before planning starts. Our study implies that inverse planning could start before OARs are approved and the final plan quality characterized by the manually drawn OARs could still be clinically acceptable. This information could be helpful for us to optimize our planning workflow and further improve efficiency in clinics.

## AUTHOR CONTRIBUTIONS

The corresponding author Chenyu Yan was responsible for the design of the study. Bingqi Guo helped explain the atlas‐based segmentation algorithm and the critical review of the article. Rahul Tendulkar contributed to all the patients’ data used in this study. Ping Xia provided valuable guidance and recommendations for the study.

## CONFLICT OF INTEREST

The authors declare that they have no conflict of Interest.

## Data Availability

The data that support the findings of this study are available on request from the corresponding author. The data are not publicly available due to privacy or ethical restrictions.
